# Supplementary material for: Identification of photoperiod-induced specific miRNAs in the adrenal glands of Sunite sheep (Ovis aries)
Source: Front Vet Sci. 2022 Jul 22;9:888207. doi: 10.3389/fvets.2022.888207 (PMC9354845; doi:10.3389/fvets.2022.888207)
Supplement: Supplementary file 1 [file Data_Sheet_1.ZIP › Supplementary file/miRDeep2 file.zip!/miRDeep2 file/Know_Structure/SP42A3/oar-mir-25.pdf]

miRBase precursor : oar-mir-25
Total read count : 16720
oar-miR-25 read count : 16635
remaining reads : 85

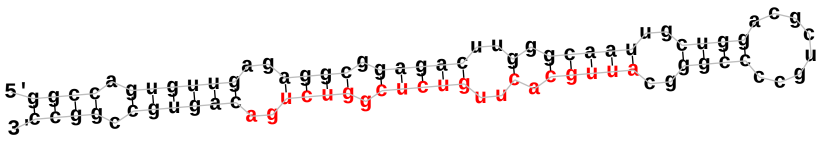

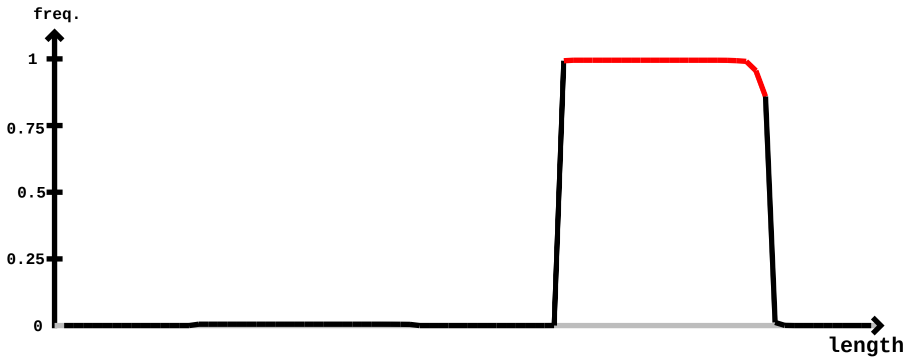

oar-miR-25

```
5'- ggccaguguugagaggcggagacuugggcaauugcuggacgcugccccgggcauugcacuugucucggucugacagugccggcc   -3'    exp
    ((((.((((((..((((.(((((..(.(((((..((((........))))..))))).)..))))).)))).)))))).))))    reads      mm         sample
    .............aggcggagacuugggcaauu...................................................    2          0          seq
    .............aggcggagacuugggcaauug..................................................    3          0          seq
    .............aggcggagacuugggcaauugc.................................................    7          0          seq
    .............aggcggagacuugggcaauugcu................................................    73         0          seq
    ..................................................gcauugcacuugucucggucuga...........    3          0          seq
    ...................................................cauugcacuugucucgg................    5          0          seq
    ...................................................cauugcacuugucucggu...............    24         0          seq
    ...................................................cauugcacuugucucgguc..............    44         0          seq
    ...................................................cauugcacuugucucggucu.............    581        0          seq
    ...................................................cauugcacuugucucggucug............    1616       0          seq
    ...................................................cauugcacuugucucggucuga...........    14135      0          seq
    ...................................................cauugcacuugucucggucugac..........    184        0          seq
    ...................................................cauugcacuugucucggucugaca.........    11         0          seq
    ....................................................auugcacuugucucggucu.............    1          0          seq
    ....................................................auugcacuugucucggucug............    3          0          seq
    ....................................................auugcacuugucucggucuga...........    28         0          seq
```
